# Renal colic caused by mycotic iliac artery aneurysm

**DOI:** 10.1259/bjrcr.20150155

**Published:** 2015-09-15

**Authors:** Anthony Cox, Shian Patel, Jeevan Kumaradevan

**Affiliations:** Department of Radiology, Whittington Hospital NHS Trust, London, UK

## Abstract

A 33-year-old female presented with acute colicky left loin-to-groin pain and microscopic haematuria, with a background of 6 months of muscle and joint pains and diplopia. A CT kidneys/ureters/bladder demonstrated fat stranding surrounding the left ureter, as it passed over the left common iliac vessels. Arterial and delayed phase imaging revealed an obstructed ureter secondary to a left common iliac artery aneurysm, later found to be mycotic. No previous descriptions of a mycotic aneurysm presenting as renal colic have been found in the literature. The diagnosis and management of infective endocarditis and mycotic aneurysm are discussed, with a review of the literature. This serves as a good example of a common presenting complaint occurring secondary to a rare and serious pathology.

## Summary

A 33-year-old female presented with acute colicky left loin-to-groin pain and microscopic haematuria, with a background of 6 months of muscle and joint pains and diplopia. A CT kidneys/ureters/bladder (KUB) demonstrated fat stranding surrounding the left ureter, as it passed over the left common iliac vessels. Arterial and delayed phase imaging revealed an obstructed ureter secondary to a left common iliac artery aneurysm, later found to be mycotic. No previous descriptions of a mycotic aneurysm causing this common presenting complaint have been found in the literature. The management of infective endocarditis (IE) and mycotic aneurysm is discussed, with a review of the literature. This serves as a good example of a common presenting complaint occurring secondary to a rare and serious pathology.

## Clinical presentation

A 33-year-old female presented to the emergency department of a district general hospital with acute onset of left loin-to-groin pain and microscopic haematuria. 1 year prior to this presentation, the patient had undergone bilateral breast augmentation with subpectoral silicone breast implants. A blood culture was performed prior to discharge for isolated pyrexia, which was positive for *Staphylo-coccus aureus.*

Over the 6 months prior to her acute presentation, she had been seen in the rheumatology and neurology outpatient departments for muscle and joint aches and an isolated episode of visual field defect/diplopia. On examination, there were no signs of peritonism. Her blood tests revealed significantly elevated C-reactive protein (CRP) levels and neutrophilia. The pain resolved with diclofenac and opiates.

## Differential diagnosis

The provisional diagnosis of renal colic was made and a CT KUB was arranged by the emergency department to examine for radiopaque renal tract calculus. Given the elevated CRP level, pyelonephritis was also considered. With a background of neurological and rheumatological symptoms, a vasculitis such as systemic lupus erythematosus could be considered, although there was no malar rash or photosensitivity.

## Imaging

**Figure 1.**
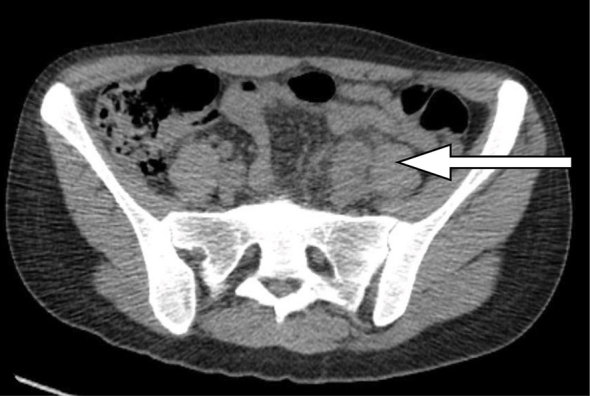
CT kidneys/ureters/bladder performed on admission to the emergency department with left renal colic. The indistinct left iliac vessels and ureter are noteworthy.

**Figure 2.**
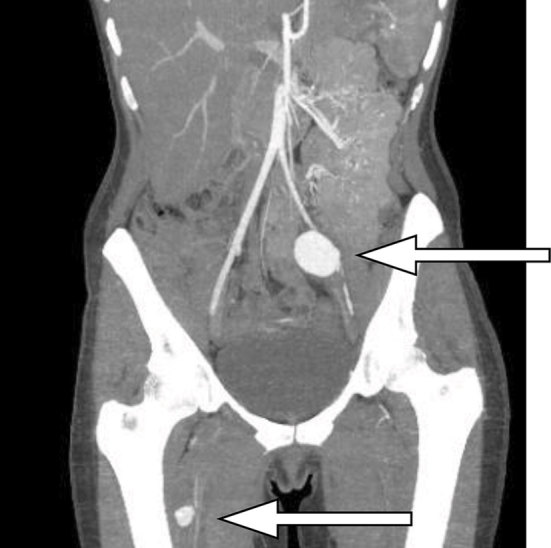
Arterial phase contrast CT (maximum intensity projection) showing left common iliac and right profunda femoris artery aneurysms.

**Figure 3.**
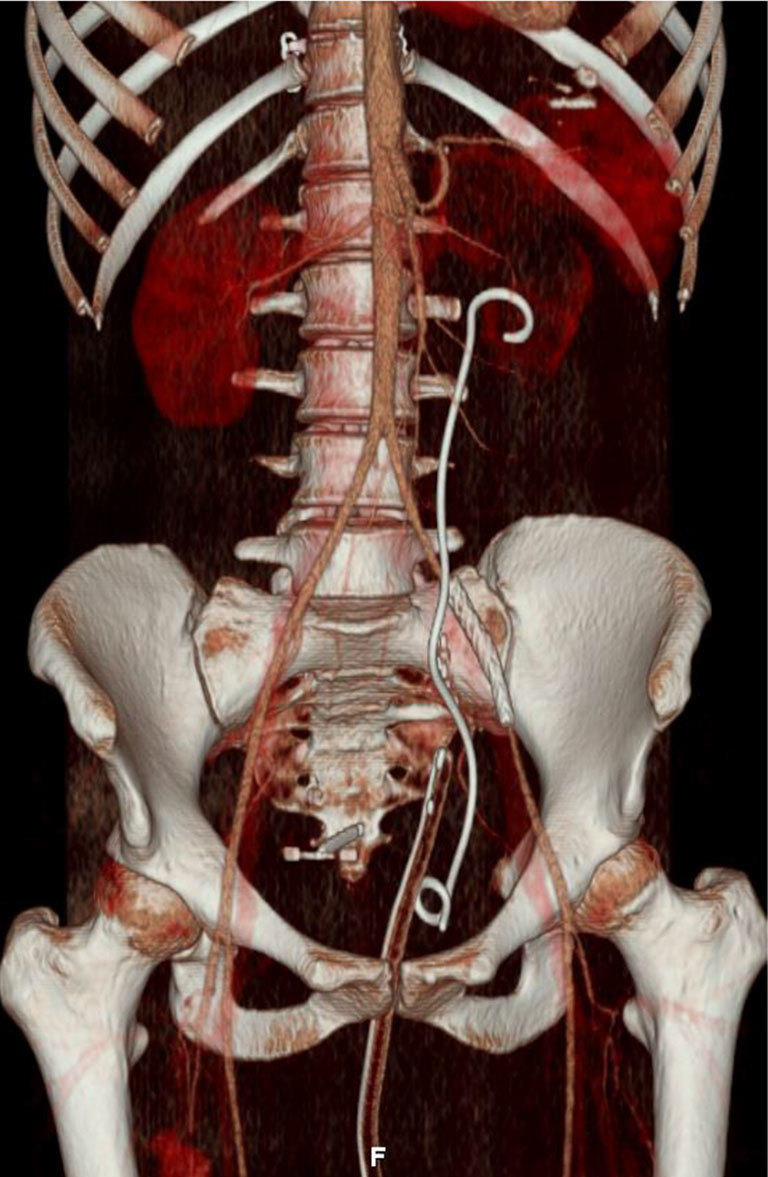
Three-dimensional arterial phase CT scan showing stent graft of the left common iliac artery aneurysm, left ureteral stent and right profunda femoris artery (prior to thrombin injection, rupture or embolization coils).

Low-dose, unenhanced CT KUB revealed (Figure 1) fat stranding and inflammation around the left ureter, as it crossed the left common iliac vessels, which were indistinct. No radiopaque calculus or other intraluminal obstruction could be seen within the ureter. The proximal ureter was distended, indicating an obstruction, but no hydronephrosis was evident. A delayed phase CT scan demonstrated an obstructed ureter at the level of the iliac vessels and the subsequent arterial phase (Figure 2) demonstrated a 3-cm common iliac artery aneurysm causing ureteric stricturing, as well as multiple aneurysms in the right profunda femoris. An intracranial CT angiogram demonstrated a right middle cerebral artery aneurysm (Figure 3). An MRI of the brain revealed a tiny foci of ischaemia affecting the left parietal and occipital lobes bilaterally, suggestive of septic emboli. Transoesophageal echocardiography (TOE) performed to examine for endocarditis revealed aortic valve vegitations.

## Treatment

Endocarditis organism eradication was initiated with long-term intravenous antibiotics. The patient underwent metallic aortic valve replacement for regurgitation and was therefore placed on wafarin, despite having multiple mycotic aneurysms elsewhere.

Owing to the risk of fistulation between the ureter and the artery, the left ureter was stented (retrograde double-J stent; Well Lead, Guangzhou, China) and a covered stent was placed across the left common iliac artery aneurysm, with an amplatzer vascular plug (St. Jude Medical, Plymouth, MN) deployed in the internal iliac artery owing to the aneurysm’s proximity to the iliac bifurcation and the risk of type II endoleak. Angiography of the medial sacral/L4 lumbar arteries demonstrated a type II endoleak, which was therefore coiled.

**Figure 4.**
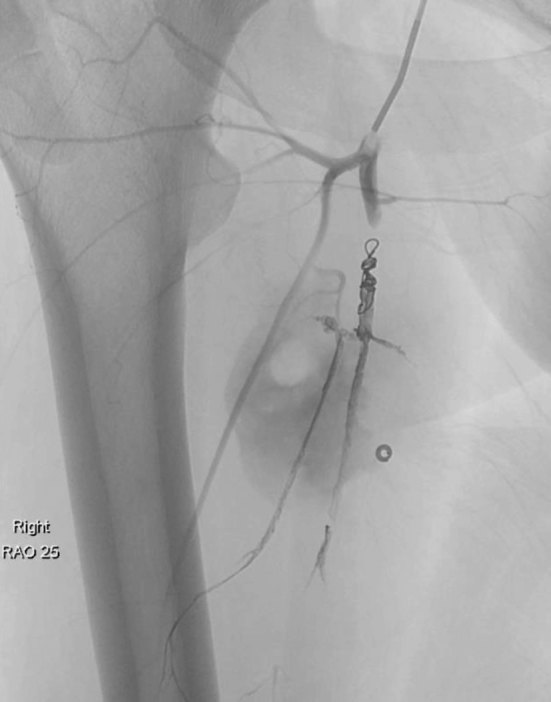
Flouroscopic image of ruptured right profunda femoris artery aneurysm after embolization with platinum coils.

The right profunda femoris artery aneurysm was injected twice with 500 units of human thrombin via a microcatheter. The patient presented 1 year later with right profunda femoris artery rupture. This was embolized with Onyx (Covidien, Plymouth, MN). Two 5 × 5.5 mm Vortex microcoils (Boston Scientific, Natick, MA) were then deployed proximal to the Onyx cast within the main profunda femoris artery (Figure 4). The patient was kept under continuous surveillance for a year and remained stable during this period.

## Discussion

### Infective endocarditis

IE is defined as an inflammation of the endocardium, most commonly affecting the valves. The aetiology is usually bacterial infection with *S*. *aureus* and streptococcus (80%). Staging, grading and classification is performed according to the modified Duke criteria. Imaging traditionally involves TOE.^1–3^

The natural history of IE affecting the prosthetic valves includes abscesses, fistulas and valve dehiscence, resulting in paravalvular regurgitation.^4^ Septic emboli and haematogenous seeding to remote sites are frequent (mostly neurological symptoms). In cases with severe or delayed diagnosis, the disease carries a high mortality rate of up to 40%.

### Mycotic aneurysms

A mycotic aneurysm is defined as an aneurysm arising from an infection of the arterial wall, usually bacterial. They rapidly grow as a focal saccular aneurysm arising eccentrically from the arterial wall. Mycotic aneurysms only represent 0.7–2.6% of all aortic aneurysms. Mycotic aneurysms are most common in the aorta, less commonly occurring elsewhere.^5^ Pathologically, mycotic aneurysms are either primary, arising from a distant, unknown or remote source of infection, or secondary, arising from a specific source of infection. Bacterial aortitis is most commonly caused by salmonella or *S. aureus.*

On imaging, periarterial soft tissue stranding, oedema and fluid are seen on the CT images. Bacterial aortitis rarely calcifies, with the exception of syphilitic aortitis, which shows curvilinear calcifications. The infection may spread to the adjacent vertebral bodies or the psoas muscle if the aneurysm is aortic. On MRI, *T*_1_ weighted images show periaortic low signal intensity in the absence of gadolinium (Gd), with subsequent aortic and periaortic enhancement following Gd, especially evident on fat-suppressed images. On *T*_2_ weighted imaging, perianeurysmal high signal intensity is seen on fat-suppressed *T*_2_ weighted images. A contrast-enhanced MR angiogram shows a saccular aneurysm(s) arising from the aortic wall. A nuclear medicine scan shows an increased uptake of labelled leukocytes at the site of the aneurysm.^5^

Surgical resection/grafting following antibiotic therapy is the definitive treatment. Open surgical repair of infected arterial aneurysms carries a significant risk of mortality and morbidity. *In situ* graft revascularization is viable in afebrile patients or patients who have good response to preoperative antibiotic therapy.^6,7^ Extra-anatomic bypass grafting for infected infrarenal abdominal aneurysm has a similar long-term survival rate and should be considered in patients who are unsuitable for *in situ* graft revascularization; however, the postoperative complication rate is higher.^6-8^ *In situ* grafts often employ a bactericidal coating (rifampicin bonded) with an omental pedicle graft as a cover; the more recent techniques include the use of cryopreserved arterial homograft.^7,9^

Endovascular treatment remains a controversial treatment option, but is considered if aneurysmectomy is not feasible in high-risk surgical candidates, although, even in this cohort, it is crucial that the infection is treated adequately prior to stent graft placement.^10,11^ Stent graft placement provides valuable treatment of arterial fistulation, which was the feared complication in this case (fistulation between the common iliac artery and the ureter).^12,13^ The natural history of mycotic aneurysms are always fatal if left untreated, with acute rupture/haemorrhage seen in 75% of cases.^13^

## Learning points

1. Multiple mycotic aneurysms, presenting as renal colic, is rare and, to our knowledge, not previously described.
2. A high index of suspicion is essential for the diagnosis of this rare condition, as septic emboli can cause devastating sequelae and all untreated infected aneurysms eventually rupture.
3. Although aneurysmectomy and antibiotics is the treatment of choice, complicating factors such as multiple large mycotic aneurysms, acute risk of utereral–arterial fistulation and warfarinization after valve replacement prohibited this definitive treatment and necessitated immediate aneurysm exclusion by endovascular treatment.
